# Using marginal structural models to adjust for treatment drop‐in when developing clinical prediction models

**DOI:** 10.1002/sim.7913

**Published:** 2018-08-02

**Authors:** Matthew Sperrin, Glen P. Martin, Alexander Pate, Tjeerd Van Staa, Niels Peek, Iain Buchan

**Affiliations:** ^1^ Farr Institute, Faculty of Biology, Medicine and Health University of Manchester, Manchester Academic Health Science Centre Manchester UK; ^2^ Microsoft Research Cambridge UK

**Keywords:** clinical prediction models, counterfactual causal inference, longitudinal data, marginal structural models, treatment drop‐in, validation

## Abstract

Clinical prediction models (CPMs) can inform decision making about treatment initiation, which requires predicted risks assuming no treatment is given. However, this is challenging since CPMs are usually derived using data sets where patients received treatment, often initiated postbaseline as “treatment drop‐ins.” This study proposes the use of marginal structural models (MSMs) to adjust for treatment drop‐in. We illustrate the use of MSMs in the CPM framework through simulation studies that represent randomized controlled trials and real‐world observational data and the example of statin initiation for cardiovascular disease prevention. The simulations include a binary treatment and a covariate, each recorded at two timepoints and having a prognostic effect on a binary outcome. The bias in predicted risk was examined in a model ignoring treatment, a model fitted on treatment‐naïve patients (at baseline), a model including baseline treatment, and the MSM. In all simulation scenarios, all models except the MSM underestimated the risk of outcome given absence of treatment. These results were supported in the statin initiation example, which showed that ignoring statin initiation postbaseline resulted in models that significantly underestimated the risk of a cardiovascular disease event occurring within 10 years. Consequently, CPMs that do not acknowledge treatment drop‐in can lead to underallocation of treatment. In conclusion, when developing CPMs to predict treatment‐naïve risk, researchers should consider using MSMs to adjust for treatment drop‐in, and also seek to exploit the ability of MSMs to allow estimation of individual treatment effects.

## 1 INTRODUCTION

Healthcare systems worldwide face escalating pressures from more people living longer with one or more long‐term conditions. To meet this challenge, interventions must move to earlier stages of disease, slowing disease progression, thereby reducing the time consuming more expensive healthcare, and increasing quality‐adjusted life years. This change requires better targeting of limited healthcare resources; the foundation for such targeting is prediction.^1^ Clinical prediction models (CPMs) typically predict the risk of an adverse outcome (eg, heart attack) based on what is currently known about an individual (eg, covariates).^2^ Clinical prediction models can be used to support decision making about treatment initiation, facilitate the discussion of treatment risks, and underpin risk stratification analyses.

Here, we consider the development of CPMs from electronic health record data to guide the commencement of drug treatments as preventive interventions among patients at high risk of common chronic disease events, eg, statins to help prevent heart attack or stroke. To support treatment initiation decisions, the risk calculated by a CPM should apply to the patient assuming that no treatment is given.^3^ However, CPMs are typically derived using observed data where patients do receive treatment, often in a time‐dependent fashion.^4^ If time‐dependent treatments are not accounted for during CPM development, the subsequent risk predictions could be incorrect owing to misspecified covariate‐outcome associations,^5^ ie, the “treatment paradox.”^6^

Recent work has, therefore, focussed on adjusting for the effect of treatment by explicitly including baseline treatment within the modeling framework.^7^ An alternative approach is to select a treatment‐naïve cohort at baseline^8^; however, this restricts the applicability of the model to those not under treatment. Importantly, these approaches do not account for patients commencing (or changing) treatment after baseline, but before the outcome of interest, so‐called “treatment drop‐ins.”^9^ For example, QRISK3 predicts 10‐year risk of cardiovascular events conditional on baseline risk factors, which is derived in a “treatment‐naïve” cohort by removing all patients who take statins at baseline.^8^ However, this means that patients who contribute to the 10‐year risk calculation may commence statins during the 10‐year follow‐up, making the interpretation of a 10‐year risk derived from such a model difficult.^10^ For example, a patient's predicted risk of lower than 10% may be driven by similar patients in the derivation cohort taking statins shortly after baseline.

The literature on accounting for treatment drop‐in is sparse. One possible approach is to restrict analysis to a population with no treatment drop‐ins, which could be achieved by selecting either a historical cohort before treatment was available, or selecting only patients who do not commence treatment during follow‐up. However, the former approach is likely to produce a model that is not relevant to current practice,^11^ while the latter approach is subject to selection bias. A refinement might be to censor patients when they commence treatment, but this would implausibly assume that treatment drop‐ins are uninformative with respect to risk factor progression after baseline (ie, treatment drop‐in depends only on baseline risk factors).^12^ A further possible approach is to estimate risk based on very large cohorts over very short time periods, thereby minimizing the potential for treatment drop‐in.^9^ Nevertheless, low probability short‐term risks may be of less clinical relevance, and extrapolating these to long‐term risks requires strong assumptions. Alternatively, Simes et al used a penalized Cox approach for treatment drop‐in in the context of a clinical trial with differential treatment drop‐in by trial arm.^13^ Here, one adjusts the event rates for the assumed effect of the “dropped in” treatments in a time‐updated fashion. The use of external estimates of the effect of the dropped in treatments avoids the issue of selection bias,^14^ but does require an assumption of transferability of effect size across contexts.^15^

In this paper, we ask the question, can combining marginal structural models (MSMs) with predictive modeling approaches generate CPMs that appropriately estimate risk in a variety of treatment regimens? Marginal structural models “subtract” the effect of both current and future treatment use, appropriately adjusting for the association between treatment drop‐in and risk factor progression postbaseline. Importantly, MSMs estimate the difference in risk for a patient who receives treatment under different regimes (ie, the causal effect of treatment under the counterfactual framework). In contrast, the aforementioned modeling techniques described cannot be used in this way since they do not explicitly consider counterfactuals.^16^ In practice, CPMs are often used in a counterfactual manner,^17^ so if such an interpretation were possible, this would be useful.

## 2 METHODS

### 2.1 Marginal structural models within the CPM framework

**Figure 1:**
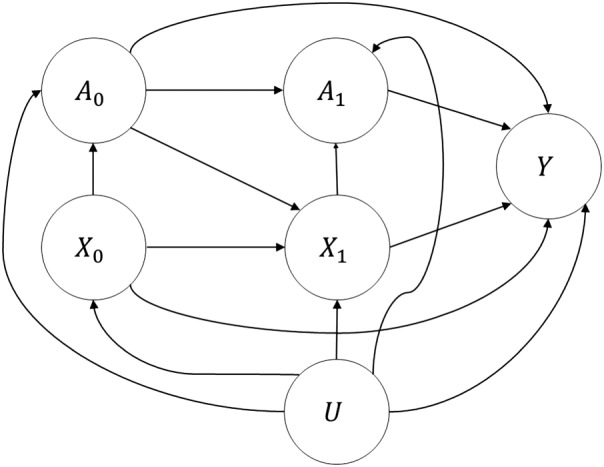
Causal diagram for simplified example

To formulate and illustrate the ideas, we consider a simplified causal model, as illustrated in Figure 1, considering a single treatment and two time steps. For causal modeling, we work in the potential outcomes framework.^18^ We suppose that at time 0, we wish to estimate a patient's risk of a future outcome, *Y*, given their baseline risk factors measured at or just before time 0, *X*_0_. The prediction will be used to support the decision regarding intervention *A*_0_, applied at or just after time 0. We use time 1 to represent future values of risk factors and intervention levels, which are *X*_1_ and *A*_1_, respectively, and acknowledge possible unmeasured confounding *U*. Of course, future values of the risk factors, *X*_1_, are unavailable at time 0 when the prediction is being made. In general, there are likely to be many future times 1, 2, …, *K*; for example, in computing 10‐year risk of an outcome, we may consider *K* = 9 annual reviews of risk factors and treatments. Let $\bar{A}=\left(A_{0},A_{1},\text{⋯},A_{K}\right)$ denote the treatment history, and let $\bar{X}=\left(X_{0},X_{1},\text{⋯},X_{K}\right)$. Let ${\bar{X}}_{-0}=\left(X_{1},\text{⋯},X_{K}\right)$; let $\bar{A}=\bar{0}\;$ mean no treatment received at any time, and write ${\bar{A}}_{k-1}$ to mean the treatment history up to time *k* − 1. We could also consider multiple treatments, where each *A*_*k*_ is a vector of length *m* to represent *m* treatments. One could regard this as a partially observable Markov decision process.^19^

A CPM seeks, at time 0, to determine future risk of Y = 1, using the information currently available (ie, X_0_, and potentially A_0_). There are various ways we could consider handling treatment, which correspond to different causal estimands. We use the notation that Z(B = b) refers to the value of Z given that we intervene to set B to value b.

E1. E[Y ∣ X_0_]: the risk of Y, disregarding the intervention.
E2. E[Y(A_0_ = 0) ∣ X_0_]: the risk of Y given that we do not intervene now, and may or may not intervene in the future.
E3. $E[Y\left(\bar{A}=\bar{0}\right)|X_{0}$]: the risk of Y given that we do not intervene now, nor do we intervene in the future.
E4. E[Y(A_0_ = 1) ∣ X_0_]: the risk of Y given that we intervene now, and may or may not intervene in the future.
E5. $E[Y\left(\bar{A}=\bar{1}\right)|X_{0}$]: the risk of Y given that we intervene now, and continue to intervene in the future.

Most existing prognostic models provide estimates of E1 or E2. In the absence of unmeasured confounding, U, the observed risk E[Y ∣ X_0_, A_0_ = 0] is a valid estimator for E2. However, calculating the risk based on not intervening immediately may provide inappropriate reassurance, since a low risk may be driven by data from patients who commence the intervention shortly after time 0.

Therefore, E3 is the treatment‐naïve risk that is truly of interest to support the decision of whether to intervene. Even in the absence of unmeasured confounding, E3 is challenging to estimate, since standard regression estimators are not valid whether or not we condition on ${\bar{X}}_{-0}.$ 20 If we do not condition, the estimate $E[Y|X_{0},\bar{A}=\bar{0}$] is prone to a “healthy survivor” bias since patients in the development cohort who remain untreated throughout are likely to have future risk factors that are better than similar patients who initiate treatment.

Conversely, if we do condition, an estimate of the form $E[Y|\bar{X},\bar{A}=\bar{0}$] will mask some of the benefits of the intervention since these manifest in ${\bar{X}}_{-0}$, not to mention that the model would be useless in practice since ${\bar{X}}_{-0}$ is unknown at time 0.

The solution to estimating risks of the form E3 is the MSM,^20^, ^21^ which applies a weighting to the population to “break” the arrows from $\bar{X}$ to $\bar{A}$, and provides a valid estimator for E3 in the absence of residual confounding. In the usual application of MSMs for causal inference, we would condition only on variables that moderate the treatment effect. In the CPM case, we also condition on variables that have only a prognostic effect (ie, those that do not modify the effect of treatment). Hence, we would like to fit a model within strata of X_0_.

The proposed approach then proceeds as follows.

1. Calculate stabilized weights for each individual i, using the formula
  $$sw_{i}=\prod_{k=0}^{K}{\left({\hat{p}}_{\mathrm{ki}}^{*}\right)}^{a_{\mathrm{ki}}}{\left(1-{\hat{p}}_{\mathrm{ki}}^{*}\right)}^{1-a_{\mathrm{ki}}}/\left\{\prod_{k=0}^{K}{\left({\hat{p}}_{\mathrm{ki}}\;\right)}^{a_{\mathrm{ki}}}{\left(1-{\hat{p}}_{\mathrm{ki}}\right)}^{1-a_{\mathrm{ki}}}\right\}.$$
  Here, ${\hat{p}}_{\mathrm{ki}}^{*}$ is the estimated predicted value from a model for $\text{logit}\;P\;\left[A_{k}=1|{\bar{A}}_{k-1},X_{0}\right],$while ${\hat{p}}_{\mathrm{ki}}$ is the estimated predicted value from a model for $\text{logit}\;P\;\left[A_{k}=1|{\bar{A}}_{k-1},{\bar{X}}_{k}\right]$. This follows the classic development of calculating weights for a MSM,^20^ besides that, to reiterate, X_0_ comprises all baseline variables that are prognostic for Y, rather than only the effect modifiers.
2. Using the derived stabilized weights, fit the model
  $$\text{logit}\;P\;\left[Y=1\right|X_{0},\bar{A}]=\beta_{0}+\beta_{X}X_{0}+\sum_{k=0}^{K}\left(\beta_{A_{k}}A_{k}+\beta_{A_{k}X}A_{k}X_{0}\right).$$
  The model allows any of the variables in X_0_ to modify the effect of treatment. We may fix by design some (or all) of the elements of $\beta_{A_{k}X}$ to 0. Similarly, a subset of X_0_ may be considered by fixing some β_X_ to 0.

Succinctly, the strategy is to adjust for variables that are available at baseline and are to be used as predictors, plus treatment strategy at baseline and in the future, and then to reweight for all remaining variables that might be on the treatment causal pathways. Generating a CPM in this manner allows us not only to estimate treatment‐naïve risk that accounts for treatment drop‐in, but also to estimate the (counterfactual) causal effect of treatment for a patient with given baseline risk factors.

### 2.2 Simulation design: overview

**Figure 2:**
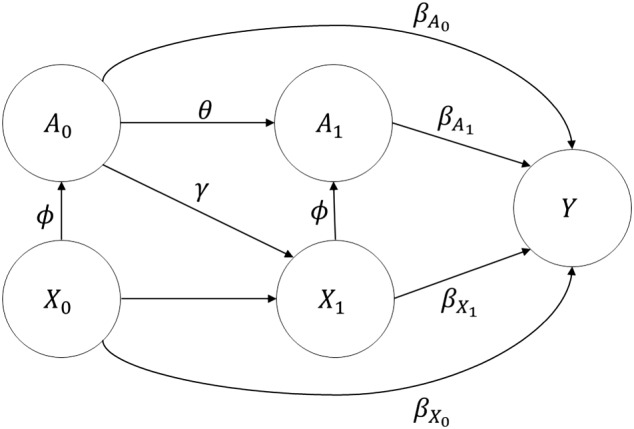
Causal diagram and parameters of the data‐generating mechanism

We designed a simulation study to demonstrate the properties of the proposed method, compared with current approaches of handling treatment when developing CPMs. Specifically, the aim was to investigate the extent of bias in predicted risk by failing to account for treatment drop‐ins. For simplicity of illustration, we again consider a scenario where we have one treatment option and two timepoints, ie, time 0 when the predictions are to be made, and a “future” time 1. At each timepoint, we record information on a single time‐varying continuous covariate and a binary treatment indicator (also time varying) (Figure 2). While, in practice, CPMs include more than one risk factor, one can imagine that the single covariate is a summary of multiple risk factors; this follows similar reasoning to previous simulation studies.^7^ Both the covariate and the treatment indicator have a prognostic effect at each timepoint on a binary outcome, where treatment reduces risk of outcome and higher values of the covariate increase risk. For example, one could imagine that Y represents a cardiovascular event, $\bar{X}$ is cholesterol (HDL ratio), and $\bar{A}$ statins.

Several simulation scenarios were considered, representing a mixture of randomized controlled trials (where treatment allocation at baseline is independent of the continuous covariate) and observational studies (where treatment allocation at each timepoint depends on the continuous covariate) under different causal pathways (see the following and Table 1 for details). Within the generated data, we fit a model simply ignoring treatment, a treatment‐naïve model (fitted on all patients without treatment at baseline), a model incorporating baseline treatment as a predictor, and the MSM.

**Table 1:** Description and parameter formulisation across each simulation scenario

Simulation Scenario	Description	Parameter values
RCT: 10% dropout†	A randomized controlled	*ϕ* = 0
	trial with treatment	*α*_0_ *s*. *t*. *P* (*A*_0_ = 1) = 0.5
	randomly allocated to 50%	$\pi_{i,A_{1}}=\theta a_{0i}$
	of the population at baseline,	*θ* = 0.9
	with 10% treatment dropout.	*α*_*Y*_ s. t. *P* (*Y* = 1) = 0.2
		$\beta_{A_{0}}=\beta_{A_{1}}=\log\left(0.5\right)$
		$\beta_{X_{0}}=\beta_{X_{1}}=\log\left(1.5\right)$
Observational: 50%	An observational study	*ϕ* = log (2)
treated	where 50% of the population	*θ* = log (2)
	have treatment.	*α*_*j*_ : *j* = 0, 1 s. t. *P* (*A*_*j*_ = 1) = 0.5
		*α*_*Y*_ s. t. *P* (*Y* = 1) = 0.2
		$\beta_{A_{0}}=\beta_{A_{1}}=\log\left(0.5\right)$
		$\beta_{X_{0}}=\beta_{X_{1}}=\log\left(1.5\right)$
Observational: 20%	An observational study	*ϕ* = log (2)
treated	where 20% of the population	*θ* = log (2)
	have treatment.	*α*_*j*_ : *j* = 0, 1 s. t. *P* (*A*_*j*_ = 1) = 0.2
		*α*_*Y*_ s. t. *P* (*Y* = 1) = 0.2
		$\beta_{A_{0}}=\beta_{A_{1}}=\log\left(0.5\right)$
		$\beta_{X_{0}}=\beta_{X_{1}}=\log\left(1.5\right)$

† : results from across a range of percentage dropouts (values of *θ*) gave similar results as those for the RCT: 10% dropout scenario and so are omitted. They are available on request.

The predictive performance of each modeling technique was calculated in two “test” data sets that were independent of the data used to derive each model (details as follows). For each simulation scenario, the relationship between *A*_0_ and *X*_1_ was controlled through the value of *γ*, which was varied through (−3, −2.5, −2, −1.5, −1, −0.5, 0). Since we assumed that treatment was effective and the covariate increased risk of outcome, we did not consider positive values of *γ*. In our example, *γ* could represent the cholesterol‐lowering effect of statins. For each value of *γ*, we repeated the simulation across 1000 iterations. The predicted performance was averaged across iterations and empirical standard errors were calculated. The simulation was implemented in R version 3.4.0,^22^ and the code is available as an online data supplement.

### 2.3 Simulation design: data‐generating mechanism

Within each iteration of a given simulation scenario, data of *N* = 10 000 observations were generated, acting as “development” data, on which one is interested in deriving a CPM. The steps of the data‐generating mechanism were the following.

1. Simulate *N* realizations of *X*_0_ ∼ *N*(0, 1).
2. Simulate *N* realizations of $A_{0}∼\text{Binomial}\left(\pi_{i,A_{0}}\right)$, where
  $$\pi_{i,A_{0}}=\frac{\exp\left(\alpha_{0}+\phi x_{0i}\right)}{1+\exp\left(\alpha_{0}+\phi x_{0i}\right)}.$$
3. Simulate *N* realizations of *X*_1_ ∼ *N*(*X*_0_ + *γA*_0_, 1).
4. Simulate *N* realizations of $A_{1}∼\text{Binomial}\;\left(\pi_{i,A_{1}}\right)$, where
  $$\pi_{i,A_{1}}=\left\{\begin{array}{cc} \frac{\exp\left(\alpha_{1}+\phi x_{1i}+\theta a_{0i}\right)}{1+\exp\left(\alpha_{1}+\phi x_{1i}+\theta a_{0i}\right)} & ,\;\text{if simulating an observational study}\\ \theta a_{0i}\;\text{for}\;\theta \in \left[0,1\right], & \text{if simulating}\;a\;\mathrm{RCT}. \end{array}\right.$$
5. Simulate *N* realizations of *Y* ∼ Binomial(π_*i*, *y*_), where

$$\log\left(\frac{\pi_{i,y}}{1-\pi_{i,y}}\right)=\alpha_{Y}+\beta_{X_{0}}x_{0i}+\beta_{X_{1}}x_{1i}+\beta_{A_{0}}a_{0i}+\beta_{A_{1}}a_{1i}.$$

The values of the aforementioned parameters across simulation scenarios are given in Table 1. Across all simulation scenarios, we assumed that the covariate increased the risk of outcome (ie, $\beta_{X_{0}}=\beta_{X_{1}}=\log\left(1.5\right)$), the treatment decreased risk of outcome ( $\beta_{A_{0}}=\beta_{A_{1}}=\log\left(0.5\right)$), and the mean event rate for the outcome, *Y*, was set at 20%. The first scenario (denoted “RCT: 10% dropout”) aims to mimic development of a CPM within a randomized controlled trial, in which treatment was randomly allocated to 50% of observations at baseline and independent of their baseline covariate (*ϕ* = 0). Here, we assumed that 10% of those treated at baseline were untreated at timepoint one (ie, 90% remained treated throughout, with *θ* = 0.9), and that untreated patients at baseline remained untreated at timepoint one. We conducted sensitivity analyses across a range of RCT *θ* values, with the results being quantitatively similar to those presented for *θ* = 0.9, and so are omitted for clarity. In contrast, the remaining two scenarios were based on observational data, in which one unit increase in *X*_0_ or *X*_1_ doubled the odds of been given treatment at the corresponding time, *ϕ* =  log(2), and those on treatment at baseline (time zero) had twice the odds of being on treatment at time one (ie, *θ* =  log(2)). Scenario 2 (denoted “Observational: 50% treated”) assumed that 50% of patients were on treatment at each timepoint, while scenario 3 (denoted “Observational: 20% treated”) lowered this to 20% of patients at each timepoint.

### 2.4 Simulation design: modeling methods and performance measures

The following models were fit within the development set, ie, a model ignoring treatment, a model developed on a treatment‐naïve cohort, a model including baseline treatment, and the MSM. The model ignoring treatment modeled the log odds of *Y* with the baseline risk factor, *X*_0_ as the only covariate (ie, $\text{logit}\left(E\left[Y\;\right|X_{0}\right])=\beta_{0}^{\prime }+\beta_{X_{0}}^{\prime }x_{0i}$); the treatment‐naïve model was similar, except that only observations with no treatment at baseline (ie, those *i* such that *a*_0*i*_ = 0) were used in model fitting. The model including baseline treatment was fit as $\text{logit}\left(E\left[Y\;\right|X_{0},A_{0}]\right)=\beta_{0}^{\prime }+\beta_{X_{0}}^{\prime }x_{0i}+\beta_{A_{0}}^{\prime }a_{0i}$. Finally, the MSM modeled the full treatment pathway and the baseline covariate as $\text{logit}\left(E\left[Y\;\right|X_{0},\bar{A}]\right)=\beta_{0}^{\prime }+\beta_{X_{0}}^{\prime }x_{0i}+\beta_{A_{0}}^{\prime }a_{0i}+\beta_{A_{1}}^{\prime }a_{1i}$, under the weighted log‐likelihood

$$l\left(\beta \right)=\sum_{i=1}^{N}{\mathrm{sw}}_{i}\left\{y_{i}\log\left(\pi_{i,Y}\right)+\left(1-y_{i}\right)\log\left(1-\pi_{i,Y}\right)\right\},$$

where *sw*_*i*_ were calculated as described. Here,

$$\begin{array}{cc} \text{logit}\left({\hat{p}}_{\mathrm{ki}}\right) & =\text{logit}\left(P\left(A_{k}=1|{\bar{A}}_{k-1},{\bar{X}}_{k}\right)\right)=\omega_{0}+\omega_{1}a_{\left(k-1\right)i}+{\text{∑}}_{j=0}^{k}\omega_{j+2}x_{\left(j\right)i}\\ \text{logit}\left({\hat{p}}_{\mathrm{ki}}^{*}\right) & =\text{logit}\left(P\left(A_{k}=1|{\bar{A}}_{k-1},X_{0}\right)\right)=\omega_{0}^{*}+\omega_{1}^{*}a_{\left(k-1\right)i}+\omega_{2}^{*}x_{0i}, \end{array}$$

with *a*_(−1)*i*_ = 0. Thus, the numerator probabilities of the stabilized weights were modeled through a logit‐linear combination of the treatment indication at the previous timepoint and the baseline covariate. The denominator probabilities were modeled as a logit‐linear combination of the previous timepoint treatment and all previous covariate information.

We generated two further independent test data sets, each of size *N* = 100 000 observations, which were used to assess performance of each modeling method. Test data set 1 was generated under the same aforementioned data‐generating mechanism described for the development data set. Test data set 2 sets *A*_0_ = *A*_1_ = 0 for all patients, but, otherwise, used the same data‐generating mechanism (Table 2). Predictive performance was assessed in terms of calibration, discrimination, and Brier score.^23^ Calibration is the agreement between the observed event rate and that expected from the model, while discrimination is the ability of the model to distinguish cases and controls. Calibration was assessed via the calibration‐in‐the‐large, and the calibration slope was estimated from a logistic regression model for the outcome with the linear predictor from a model as the only covariate (where a perfectly calibrated model will have a slope of one).^24^ Discrimination was assessed through the area under the receiver operating characteristic curve (area under the curve).

**Table 2:** Description of the performance‐settings and corresponding test datasets

Performance‐setting	Description	Test set data‐generating mechanism
Mix of Treatment	Model validation on samples	Test set 1 (*N* = 100 000):
(MT)	drawn from a similar	Generated under exactly the same
	population to the	process as the development cohort.
	development set.	
	Corresponds to estimating E1.	
No Baseline Treatment	Model validation on samples	Test set 1 (*N* = *P*(*A*_0_ = 0)×
(NBT)	drawn from a similar	100 000):
	population to the	Generated under exactly the same
	development set, but	process as the development cohort,
	restricted to those without	but restricted to examining
	treatment at baseline.	{*i* ∈ [1, *N*] : *a*_0*i*_ = 0}.
	Corresponds to estimating E2.	
No Treatment	Model validation in a	Test set 2 (*N* = 100 000):
Throughout (NTT)	population where treatment is	generated as
	withheld from all patients,	*X*_0_ ∼ *N*(0, 1)
	but where the distribution of	X_1_ ∼ *N*(*X*_0_, 1)
	covariates is similar to the	*A*_0_ = *A*_1_ = 0
	development cohort.	
	Corresponds to estimating E3.	

Across the two test data sets, three performance settings were considered (Table 2). The first, (denoted “performance setting: mix of treatment (MT)”) used test data set 1 to estimate performance, thus representing performance on (treated and untreated) samples drawn from a similar population to the development set; this was used to examine estimate E1. The second performance setting (denoted “performance setting: no baseline treatment (NBT)”) estimated performance in test data set 1, but restricted to those observations who did not receive treatment at baseline, giving an indication of estimate E2. Finally, “performance setting: no treatment throughout (NTT)” used test data set 2 to examine the bias in the calculation of the causal effect E3 for each modeling method. Moreover, in practice, individuals might initiate treatment if the predicted (E3) risk exceeded an a priori chosen treatment threshold. Thus, to examine the impact of each modeling strategy on treatment decision making, we calculated the proportion of patients within test data set 2 where the predicted risk from a given modeling strategy was larger than a range of treatment thresholds from 5% to 70%.

### 2.5 Statin initiation in cardiovascular disease prevention example

To illustrate the MSM approach in a real‐world clinical example, we used data derived from the Clinical Practice Research Datalink (CPRD). The CPRD is a database of routinely collected primary care data, including approximately 7% of the UK population.^25^ The database collates coded data on patient demographics, prescription details, clinical events, and diagnoses.

We extracted a population of patients aged 25 to 84 at index date, who were registered in England between the January 1, 1998 and December 31, 2015. Here, the index date for each patient was chosen at random between the study start date (defined as the maximum of a patient's 25th birthday, one year of valid follow‐up, or January 1, 1998), and the study end date (defined as the minimum of March 3, 2016, the date of censoring, or the date of a patient's 85th birthday). If this time period did not have a positive number of days, or the patient had received a statin or had a cardiovascular disease (CVD) event prior to the randomly assigned index date, the patient was excluded. This time period is the same as that chosen in QRISK3,^8^ with the exception of choosing random index dates rather than the start of the study interval.

We defined the outcome to be first recorded diagnosis of CVD within 10 years of each patient's index date. Additionally, at the index date, we extracted the following baseline covariate information for each patient: age, sex, atrial fibrillation, chronic kidney disease (stage 4/5), type I or II diabetes, ethnicity, family history of coronary heart disease, hypertension, rheumatoid arthritis, and systolic blood pressure. These variables represent ***X***_0_, and were each reviewed annually, up to 10 years post index date (representing ***X***_1_, …, ***X***_9_). Missing data occurred for systolic blood pressure; we imputed this as the mean of the future observed values, and missing annual updates were imputed using a last observation carried forward approach within each patient. Patients who had no observed systolic blood pressure throughout 10‐year follow‐up were removed from the analysis.

Time from index date to prescription of first statin was available for all patients. We defined annual binary indicators of statin use (ie, *A*_0_, *A*_1_, …, *A*_9_) as 1 if (i) a given patient had started statins prior to year *k* for *k* = 0, …, 9, and (ii) the statin initiation occurred at least one year prior to any subsequent CVD event for a given patient. We did not model for statin discontinuation after first prescription.

Details of fitting time‐to‐event models under stabilized weights have been given previously.^21^ Explicitly, we fitted a treatment‐naïve model and an MSM as two pooled logistic regression models that treated each person‐year prior to either a CVD event, censoring, or 10‐year follow‐up (which ever occurred first) as an observation. The MSM included a year‐specific intercept (fitted as a restricted cubic spline), the baseline covariates (ie, ***X***_0_), and an indicator of statin use at the previous year. A further advantage of the MSM framework is that right censoring due to loss of follow‐up in CPRD could be adjusted for by conceptualizing the censoring as a second time‐varying treatment.^20^, ^21^ Thus, the MSM was estimated under a weighted likelihood with the stabilized weights accounting for treatment drop‐in and censoring; the models used to estimate the stabilized weights were also fitted through pooled logistic regression models.^21^ In contrast, the treatment‐naïve model was estimated via standard maximum likelihood and only included the year‐specific intercept (fitted as a restricted cubic spline) and the baseline covariates.

Calibration‐in‐the‐large was assessed in those patients who had no prescription of statins (at any time) to mimic estimating E3. Additionally, we calculated the proportion of patients with a predicted 10‐year CVD risk from each model greater than a range of treatment threshold values (ie, proportion treated), similar to that described in the simulation study.

## 3 RESULTS

### 3.1 Simulation study

#### 3.1.1 Calibration

**Figure 3:**
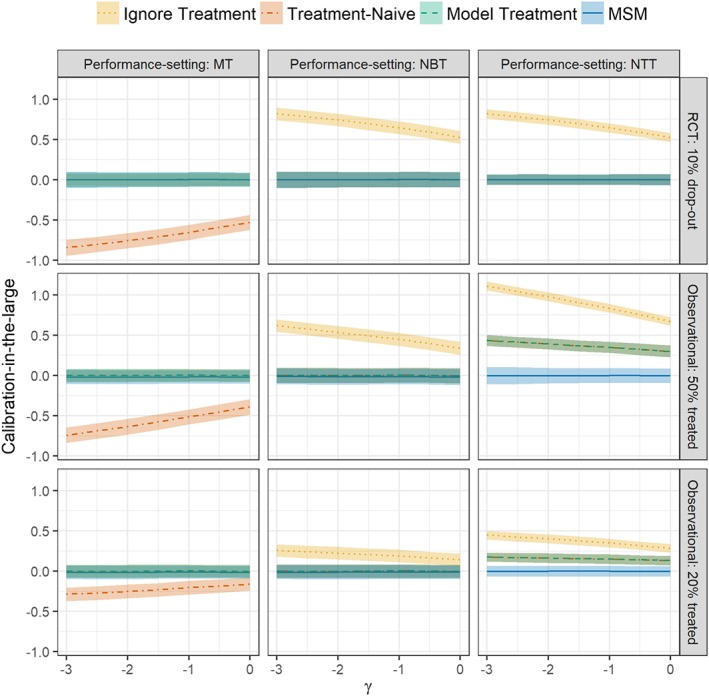
Calibration‐in‐the‐large in each simulation scenario (rows), across all performance‐settings (columns), and values of  γ (the cholesterol lowering effect of statins). In performance settings no baseline treatment (NBT) and no treatment throughout (NTT), the calibration‐in‐the‐large for the treatment‐naïve model and the model treatment is indistinguishable. MSM, marginal structural model; MT, mix of treatment [Colour figure can be viewed at http://wileyonlinelibrary.com]

Within the RCT: 10% dropout simulation scenario, the model including baseline treatment and the MSM were well calibrated across all three performance settings (Figure 3). In contrast, the model ignoring treatment underestimated E2 (performance setting: NBT) and E3 (performance setting: NTT), while the treatment‐naïve model overpredicted E1 (performance setting: MT). In both observational simulation scenarios (20% treated and 50% treated), all models except the MSM provide biased estimates of E3 (performance setting: NTT), with calibration‐in‐the‐large significantly larger than zero (Figure 3); here, the underestimation was most pronounced for the model that ignored treatment. Since the MSM can include the full treatment pathway, this model had calibration‐in‐the‐large close to zero across all values of *γ*. The bias in estimating E3 by ignoring treatment drop‐in decreased as the proportion of treated observations at each timepoint decreased.

The RCT: 10% dropout simulation scenario demonstrated calibration slopes not significantly different from one across all models except the model ignoring treatment in performance setting: NBT and performance setting: NTT (Supplementary Figure 1). In contrast, the calibration slope for the model ignoring treatment, the treatment‐naïve model and the model including baseline treatment was significantly above one in Observational: 50% treated and Observational: 20% treated simulation scenarios. This indicated that, in these observational circumstances, the coefficient of *X*_0_ in all models apart from the MSM was too low.

#### 3.1.2 Discrimination and Brier score

The discrimination of all models for simulation scenario RCT: 10% dropout were identical across performance settings: NBT and NTT (Supplementary Figure 2). For performance setting MT, the MSM resulted in the highest discrimination and lowest Brier score across all values of *γ*, with all models converging when *γ* = 0 (Supplementary Figure 2). This is likely the effect of the MSM model being able to incorporate the full treatment pathway (ie, adjusts for both *A*_0_ and *A*_1_). The area under the curve and Brier score were quantitatively similar in both observational simulation scenarios to those in the RCT scenario**,** and so are omitted for clarity.

#### 3.1.3 Treatment decision making

**Figure 4:**
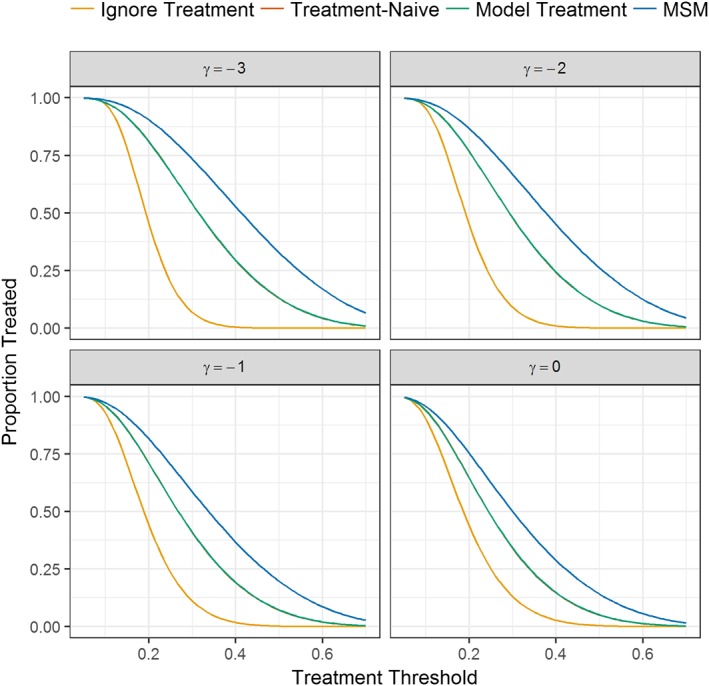
Proportion of patients in the Observational: 50% treated simulation scenario who would initiate treatment at baseline if their predicted risk given no current or future intervention exceeded a given treatment threshold. Note that γ values of −2.5, −1.5, and −0.5 have been removed for clarity. The treatment‐naïve model and the model including baseline treatment are identical. MSM, marginal structural model [Colour figure can be viewed at http://wileyonlinelibrary.com]

We examined the proportion of patients who would have treatment initiated at baseline if $E\left[Y=1\;\right|X_{0},\bar{A}=0]\;$ exceeded a given treatment threshold; Figure 4 depicts the results obtained from the Observational: 50% treated simulation scenario. Given that only the MSM provides valid estimates of E3, we take this to be the reference and find that the model ignoring treatment, the treatment‐naïve model, and the model including baseline treatment all underallocated treatment. For example, when *γ* = 0 and taking a 40% treatment threshold, the proportion of patients allocated to treatment was 2.9%, 14.9%, 15.1%, and 29.2%; for the model ignoring treatment, the treatment‐naïve model, the model including baseline treatment, and the MSM, respectively (Figure 4). Similar results were obtained across the other simulation scenarios (Supplementary Figures 3 and 4).

### 3.2 Statin initiation in CVD prevention example

A total of *n* = 3 630 818 patients were extracted from CPRD, of which *n* = 656 006 had no observed systolic blood pressure throughout follow‐up and were removed from the analysis. Hence, the final analysis sample was *n* = 2 974 812, which included 12 144 193 person‐years of follow‐up. The Kaplan‐Meier 10‐year CVD event rate was 9.15%, with an incidence rate of 8.06 per 1000 person‐years. Within the analysis sample, *n* = 170 249 (5.72%) had a prescription of first statin after index date but before either 10‐year follow‐up or at least one year prior to a CVD event occurring within 10 years.

The coefficient estimates for the baseline covariates were similar between the treatment‐naïve model and the MSM (Table 3). However, the MSM shows that statins significantly reduce the odds of a subsequent CVD event, which is not captured within the treatment‐naïve model. In the subset of patients in the analysis sample who did not have a statin “treatment drop‐in,” calibration‐in‐the‐large was 0.110 (95% CI: 0.103, 0.116) for the treatment‐naïve model, and 0.003 (95% CI: −0.004, 0.010) for the MSM; this is reflected in the calibration plots (Supplementary Figure 5) and the calibration slopes and c‐statistics (Supplementary Table 1). Hence, the MSM accurately predicted risk E3, while the treatment‐naïve model significantly underpredicted risk since it does not account for the risk lowering effect of statin use. Correspondingly, the proportion of patients with a predicted risk from the treatment‐naïve model that exceeded a given treatment threshold was lower than that from the MSM (Supplementary Figure 6). For example, a treatment threshold of >10% for the 10‐year predicted CVD risk resulted in 22.6% of patients starting treatment based on the treatment‐naïve model and 26.2% of patients based on the MSM.

**Table 3:** Parameter estimates from the Clinical Practice Research Datalink example estimated from the treatment‐naïve model and the marginal structural model (MSM). All of the variables are those extracted at baseline (ie, the index date). Note that both models include a year‐specific intercept fitted as a restricted cubic spline

Variable	Treatment Naïve Model (SE)	MSM (SE)
Statin Initiation	N/A	−0.1002 (0.0103)
Female	−0.5566 (0.0066)	−0.5331 (0.0062)
Age	0.0711 (0.0003)	0.0723 (0.0002)
Atrial Fibrillation	0.4827 (0.0156)	0.4353 (0.0148)
Chronic Kidney Disease (stage 4/5)	0.3646 (0.0352)	0.3612 (0.0318)
Type I diabetes	0.7307 (0.0585)	0.5859 (0.0564)
Type II diabetes	0.5967 (0.0138)	0.5414 (0.0128)
Ethnicity		
White or not stated	Reference	Reference
Asian	−0.0840 (0.0700)	−0.1936 (0.0679)
Bangladesh	0.1343 (0.1557)	0.1191 (0.1504)
Black	−0.6454 (0.0568)	−0.7092 (0.0536)
Chinese	−0.7970 (0.1629)	−0.7965 (0.1553)
Indian	−0.0250 (0.0498)	0.0787 (0.0440)
Mixed	−0.6138 (0.1287)	−0.6547 (0.1229)
Other	−0.3416 (0.0758)	−0.2489 (0.0674)
Pakistani	0.3351 (0.0794)	0.3109 (0.0766)
Family history of coronary heart disease	0.1391 (0.0088)	0.1172 (0.0082)
Hypertension	0.1390 (0.0078)	0.2553 (0.0072)
Rheumatoid arthritis	0.4200 (0.0237)	0.4072 (0.0220)
Systolic blood pressure	0.0109 (0.0002)	0.0104 (0.0002)

SE = standard error.

## 4 DISCUSSION

This paper introduces the concept of embedding CPMs within a counterfactual causal framework, using MSMs to adjust for treatment drop‐in, thereby better reflecting real‐world healthcare. This allows for estimation of treatment‐naïve risk that appropriately adjusts for treatment drop‐in. Moreover, we can estimate causal effects of treatment. The application of sophisticated counterfactual modeling, specifically the MSM, is novel.

Our simulation study shows that the common practice of simply ignoring time‐dependent treatment in CPM development provides biased outcome risk estimates in untreated individuals. Although including baseline treatment within the model provided some protection from this, only the MSM resulted in valid risk estimates, given no current or future intervention. Since CPMs are often used in the context of stop‐go clinical decision making regarding treatment, these results demonstrate that current approaches to developing CPMs are ill‐suited to common uses and provide misspecified covariate‐outcome associations in the presence of (time‐dependent) treatment. Failing to account for treatment drop‐ins led to significant underprediction in risk E3 and a corresponding underallocation to treatment. While the literature on handling treatments in CPM development is sparse, the results from this paper support those of previous studies.^7^, ^9^ As reported previously, within a simple two‐armed randomized controlled trial (with no treatment drop‐ins), all of the modeling strategies except ignoring treatment provided valid estimates of E3.^7^ Nevertheless, observational data sets are needed to capture the high variability in treatment initiation, adherence, and duration that occur in practice. While explicitly modeling baseline treatment is preferred to modeling within a treatment‐naïve cohort,^7^ the current study suggests that CPMs need to be framed within a counterfactual causal framework to truly support using them in treatment initiation settings. To the best of our knowledge, this is the first study to propose such a counterfactual framework for developing a CPM.

Postdevelopment CPMs need to be validated in samples similar to (internal validation) and distinct from (external validation) the development cohort.^26^ Performance setting MT in the current simulation study aimed to represent an internal validation of models within a cohort driven by the same underlying processes and with the same ratio of treated to nontreated observations. In such a situation, the treatment‐naïve modeling method was miscalibrated, which is unsurprising given that performance setting MT tests this model in both treated and nontreated observations, and agrees with previous findings.^27^ However, poor performance can be expected if models ignoring treatment or only modeling baseline treatment are then applied/validated in treatment‐naïve populations (performance setting: NTT). Importantly, all published CPM validation studies, whether internal or external, focus on the model's ability to estimate E2. If aiming to guide treatment initiation, one needs to assess the ability to estimate E3. Here, the MSM was well calibrated in all circumstances we considered since it can include the full treatment pathway. Based on such findings, we recommend that MSMs be used to develop CPMs where treatment drop‐ins are expected.

We acknowledge some limitations. First, we assume no residual confounding. Particularly, when using routinely collected observational data for causal inference, sensitivity analyses to explore residual confounding are advisable.^28^ Second, the requirements for building a deployable CPM would need more careful consideration. For example, considering the implications of introducing a causal structure on model performance and validity. Third, we note that using a CPM is itself an intervention, suggesting that a metamodel with rapid feedback may be required to understand how the use of the CPM may be changing patient care.^29^ Fourth, we have only considered a single treatment. In principle, the extension to multiple treatments sits within the methodology; although, model complexity may become an issue. Fifth, we have not modeled interaction between treatment and prognostic factors; although, it is straightforward to do this within the proposed framework.^30^, ^31^ Sixth, in our statin example, we did not explicitly model statin discontinuation, doing so would allow more detailed counterfactual modeling of treatment histories and allow us to determine the optimal time to commence statins given the risk of subsequent discontinuation.^32^ Finally, in routinely collected observational data, risk factors may be observed at different times and are likely to be subject to informative observation and missingness^33^ (eg, patients being measured more often when they are sicker^34^). Methods are needed to overcome such challenges within this framework.

In conclusion, we have shown that MSMs can improve treatment‐naïve risk estimation through better adjustment for treatment‐drop‐ins, avoiding a potentially serious underestimate of treatment‐naïve risk. This approach should be explored further in the development of CPMs.

## Supporting information

SIM_7913‐supp‐0001_Accounting for treatment in CPMs ‐ Supplements R2.docx

SIM_7913‐supp‐0002_Simulation study code.docx
