# Supplementary material for: Using marginal structural models to adjust for treatment drop‐in when developing clinical prediction models
Source: Stat Med. 2018 Aug 2;37(28):4142–54. doi: 10.1002/sim.7913 (PMC6282523; doi:10.1002/sim.7913)
Supplement: Supplementary file 1 — SIM_7913‐supp‐0001_Accounting for treatment in CPMs ‐ Supplements R2.docx [file SIM-37-4142-s001.docx]

Supplementary Material

Using marginal structural models to adjust for treatment drop-in when developing clinical prediction models

# Supplementary Figures

**Supplementary Figure 1:** Calibration slope in each simulation scenario (rows), across all performance-settings (columns) and values of $\gamma$. In Performance-settings “No Baseline Treatment” and “No Treatment Throughout”, the calibration slope for the treatment-naïve model and the model treatment are indistinguishable.


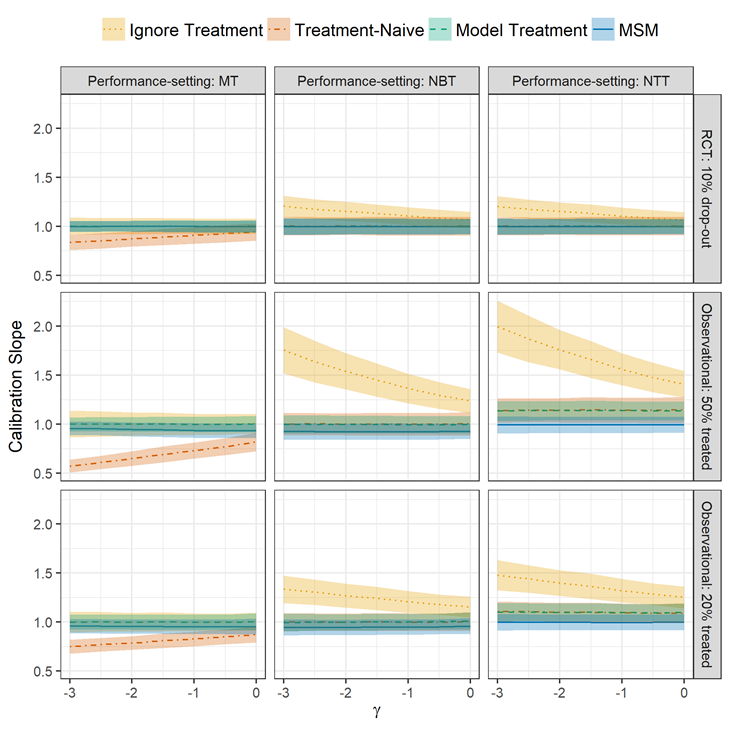


**Supplementary Figure 2:** AUC (top row) and Brier score (bottom row) across all performance-settings and values of $\gamma$ for the RCT: 10% drop-out simulation scenario. Quantitatively similar results were observed across all other simulation scenarios.


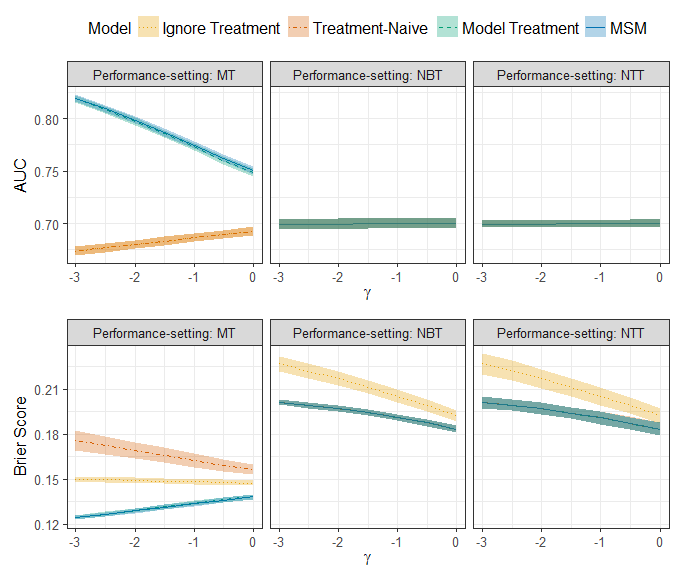


**Supplementary Figure 3:** Proportion of patients in the RCT: 10% drop-out simulation scenario who would initiate treatment at baseline if their predicted risk given no current or future intervention exceeded a given treatment threshold across each model. Note, $\gamma$ values of -2.5, -1.5 and -0.5 have been removed for clarity. The treatment-naïve model, the model including baseline and the MSM model are identical.


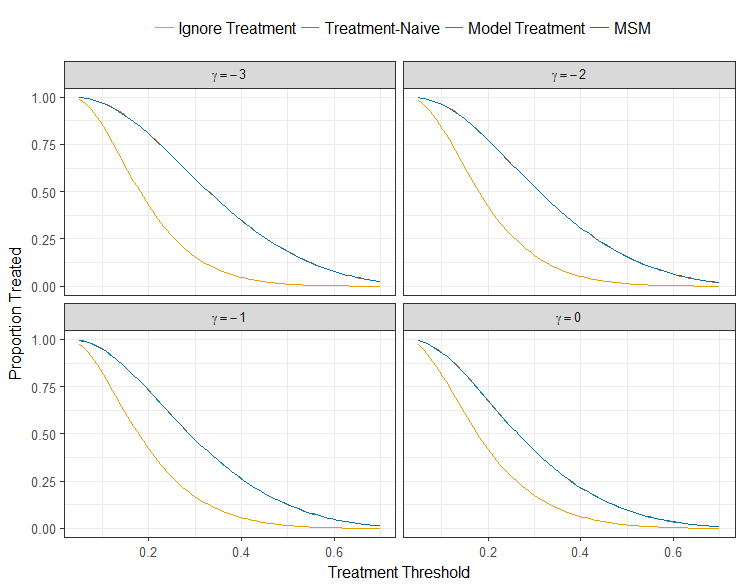


**Supplementary Figure 4:** Proportion of patients in the Observational 50% treated simulation scenario who would initiate treatment at baseline if their predicted risk given no current or future intervention exceeded a given treatment threshold across each model. Note, $\gamma$ values of -2.5, -1.5 and -0.5 have been removed for clarity. The treatment-naïve model and the model including baseline are identical.


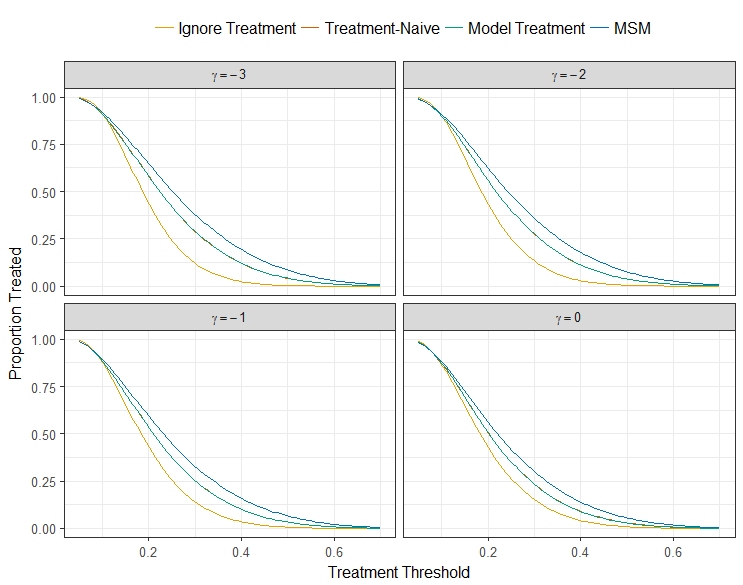


**Supplementary Figure 5:** Calibration plots for treatment-naïve model (left) and marginal structural model (right) applied to the subset of patients in the CPRD clinical example who did not have a statin ‘treatment drop-in’


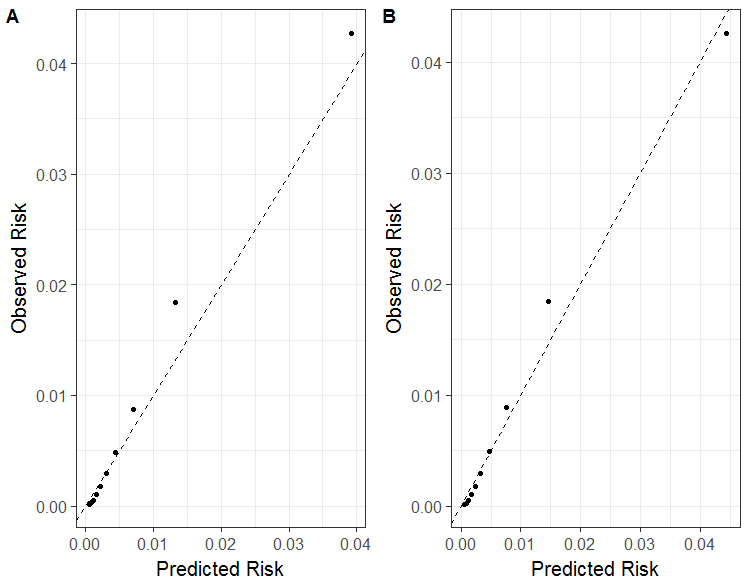


**Supplementary Table 1:** Calibration and discrimination statistics applying models to the subset of patients in the CPRD clinical example who did not have a statin ‘treatment drop-in’

| Model | Calibration-in-the-large | Calibration Slope | c-statistic |
| --- | --- | --- | --- |
| Treatment naïve | 0.110 (0.103, 0.116) | 1.031 (1.026, 1.037) | 0.855 (0.854, 0.856) |
| MSM | **0.003 (-0.004, 0.010)** | **1.005 (0.999, 1.010)** | 0.855 (0.854, 0.856) |


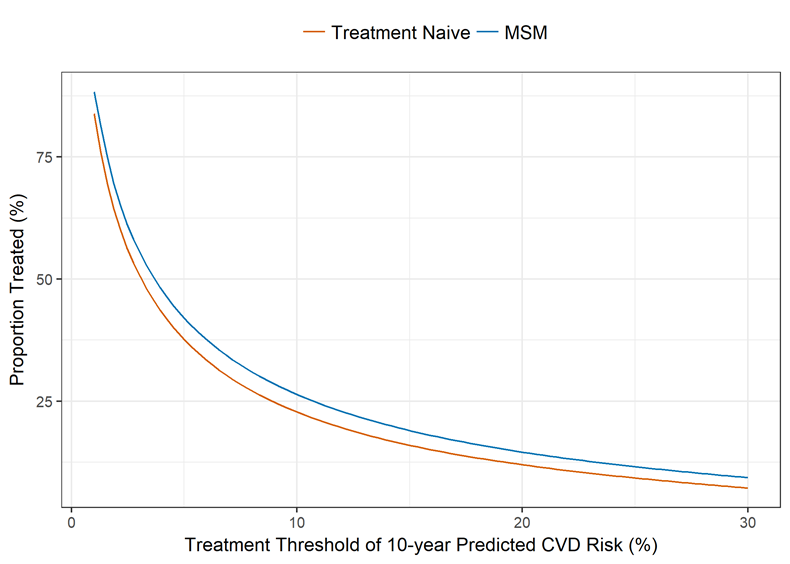


**Supplementary Figure 6:** Proportion of patients in the CPRD clinical example who would initiate treatment at baseline if their predicted risk given no current or future statin use exceeded a given treatment threshold.
