# Supplementary material for: Using marginal structural models to adjust for treatment drop‐in when developing clinical prediction models
Source: Stat Med. 2018 Aug 2;37(28):4142–54. doi: 10.1002/sim.7913 (PMC6282523; doi:10.1002/sim.7913)
Supplement: Supplementary file 2 — SIM_7913‐supp‐0002_Simulation study code.docx [file SIM-37-4142-s002.docx]

###-----------------------------------------------------------------------

## Main simulation function

###-----------------------------------------------------------------------

sim.fnc <- function(dev.samplesize, val.samplesize, iterations=1000, startingseed, filename,

betaA0, betaA1, betaX0, betaX1,

phi, theta, gamma, tau,

Eventrate_A0,

Eventrate_A1,

Eventrate_Y,

calcAUC=TRUE,

SimulationSetting=c("RCT", "Observational")){

#Input: dev.samplesize/val.samplesize = integer giving the number of observations to generate within each iteration for

# the development and test datasets, respectively.

# iterations = integer giving the number of iterations to run the simulation over - defaults at 1000

# startingseed = integer giving the starting seed for reproducibility

# filename = character name of file to store simulation results within the working directory

# betaA0 = integer of 'true' association between A0 and Prob(Y)

# betaA1 = integer of 'true' association between A1 and Prob(Y)

# betaX0 = integer of 'true' association between X0 and Prob(Y)

# betaX1 = integer of 'true' association between X1 and Prob(Y)

# phi = integer of 'true' association between X0/X1 and A0/A1

# theta = integer of 'true' association between A0 and Prob(A1); in case of RCT this is proportion of treated arm at

# baseline who remain treated at t=1.

# gamma = vector of 'true' associations between A0 and X1

# tau = integer of 'true' interaction A0*X1 on Prob(A1)

# Eventrate_A0, Eventrate_A1, Eventrate_Y = integer of the overall event rates of A0, A1 and Y, repectively.

# calcAUC = logical: should the discrimination of the modelling methods be calculated? For large val.samplesize, sig. faster if FALSE

# SimulationSetting = is the simulation aiming to mimic a randomised controlled trial or observational study?

library(plyr)

library(dplyr)

library(pROC)

set.seed(startingseed)

#Create text file to store all the estimates of interest

if(file.exists(paste(filename, ".txt", sep=""))==TRUE |

file.exists(paste("TreatmentDecision_", paste(filename, ".txt", sep=""), sep=""))==TRUE){ #no overwritting existing result files

stop("filename already exists within working directory - delete or move file to continue")

}else{

filename <- paste(filename, ".txt", sep="")

OutputNames <- c("Iteration", "A0_X1_Value", "Estimand", "SIT", "TreatNaive", "TreatmentIncluded", "MSM")

write(OutputNames, filename, append=FALSE, sep="|", ncolumns=length(OutputNames))

#Define a textfile to store treatment initiation decisions across simulations

write(c("Iteration", "A0_X1_Value", "SIT", "TN", "TI", "MSM", "TreatmentThreshold"),

file = paste("TreatmentDecision_", filename, sep=""),

append = FALSE, sep="\t",

ncolumns = length(c("Iteration", "A0_X1_Value", "SIT", "TN", "TI", "MSM", "TreatmentThreshold")))

}

#Define the situation the simulation is based on- is it representing a RCT or observational study?

SimulationSetting <- as.character(match.arg(SimulationSetting))

for(g in 1:length(gamma)){

print(paste("Starting simulation for gamma=",gamma[g], sep="")) #keep track of progress while simulation running

pb <- winProgressBar(title="Simulation Progress", label=paste("Simulation 0% Completed"), min=0, max=100, initial=0)

for(iter in 1:iterations){

#### Generate the covariate, treatment and outcome data

#Development sample

Dat <- Data.gen.fnc(samplesize = dev.samplesize,

betaA0 = betaA0, betaA1 = betaA1, betaX0 = betaX0, betaX1 = betaX1,

phi = phi, theta = theta, gamma = gamma[g], tau = tau,

Eventrate_A0 = Eventrate_A0,

Eventrate_A1 = Eventrate_A1,

Eventrate_Y = Eventrate_Y,

SimulationSetting = SimulationSetting)

Development.Dataset <- Dat$Data.gen

alpha_y <- Dat$alpha_Y

#Test set (1)

Dat <- Data.gen.fnc(samplesize = val.samplesize,

betaA0 = betaA0, betaA1 = betaA1, betaX0 = betaX0, betaX1 = betaX1,

phi = phi, theta = theta, gamma = gamma[g], tau = tau,

Eventrate_A0 = Eventrate_A0,

Eventrate_A1 = Eventrate_A1,

Eventrate_Y = Eventrate_Y,

SimulationSetting = SimulationSetting)

Test1.Dataset <- Dat$Data.gen

#Test set (2)

Test2.Dataset <- Modified.Data.gen.fnc(samplesize = val.samplesize,

betaA0 = betaA0, betaA1 = betaA1, betaX0 = betaX0, betaX1 = betaX1,

gamma = gamma[g], alpha_y = alpha_y)

#### Fit each of the models in the development dataset

SIT.mod <- glm(Y ~ X0, data=Development.Dataset, family=binomial(link="logit")) #simply ignore treatment

TreatNaive.mod <- glm(Y ~ X0, data=Development.Dataset %>% filter(A0==0), family=binomial(link="logit"))

TreatmentIncluded.mod <- glm(Y ~ X0 + A0, data=Development.Dataset, family=binomial(link="logit"))

sw <- msm.fnc(Dataset = Development.Dataset)

suppressWarnings(

MSM.mod <- glm(Y ~ X0 + A0 + A1, data=Development.Dataset, family = binomial(link="logit"), weights = sw)

) # suppress warnings of "non-integer #successes in a binomial glm!" from the weights argument

#test <- Weighted.Log.Reg(LinPreds = Development.Dataset %>% select(X0, A0, A1), Outcome = Development.Dataset$Y, sw=sw)

#browser() #test and MSM.mod should have same coefficient estimates

#### Test the models in each of the test sets

Performance1.Data <- Test1.Dataset %>%

mutate(SIT = predict(SIT.mod, newdata = ., type="response"),

TreatNaive = predict(TreatNaive.mod, newdata = ., type="response"),

TreatmentIncluded = predict(TreatmentIncluded.mod, newdata = ., type="response"),

MSM = predict(MSM.mod, newdata = ., type="response"))

Performance1 <- as.data.frame(sapply(c("SIT", "TreatNaive", "TreatmentIncluded", "MSM"),

function(x)performance.fnc(Outcomes = Performance1.Data$Y,

PredRisks = eval(parse(text=paste("Performance1.Data", x,

sep="$"))),

calcAUC = calcAUC))) %>%

mutate(Estimand = paste("Performance.E1", row.names(.), sep="_"))

Performance2.Data <- Test1.Dataset %>%

filter(A0==0) %>% #remove patients from test dataset 1 who had treatment at baseline

mutate(SIT = predict(SIT.mod, newdata = ., type="response"),

TreatNaive = predict(TreatNaive.mod, newdata = ., type="response"),

TreatmentIncluded = predict(TreatmentIncluded.mod, newdata = ., type="response"),

MSM = predict(MSM.mod, newdata = ., type="response"))

Performance2 <- as.data.frame(sapply(c("SIT", "TreatNaive", "TreatmentIncluded", "MSM"),

function(x)performance.fnc(Outcomes = Performance2.Data$Y,

PredRisks = eval(parse(text=paste("Performance2.Data", x,

sep="$"))),

calcAUC = calcAUC))) %>%

mutate(Estimand = paste("Performance.E2", row.names(.), sep="_"))

Performance3.Data <- Test2.Dataset %>%

mutate(SIT = predict(SIT.mod, newdata = ., type="response"),

TreatNaive = predict(TreatNaive.mod, newdata = ., type="response"),

TreatmentIncluded = predict(TreatmentIncluded.mod, newdata = ., type="response"),

MSM = predict(MSM.mod, newdata = ., type="response"))

Performance3 <- as.data.frame(sapply(c("SIT", "TreatNaive", "TreatmentIncluded", "MSM"),

function(x)performance.fnc(Outcomes = Performance3.Data$Y,

PredRisks = eval(parse(text=paste("Performance3.Data", x,

sep="$"))),

calcAUC = calcAUC))) %>%

mutate(Estimand = paste("Performance.E3", row.names(.), sep="_"))

Results <- bind_rows(Performance1, Performance2, Performance3) %>%

mutate("Iteration" = iter,

"A0_X1_Value" = gamma[g]) %>%

select(Iteration, A0_X1_Value, Estimand, SIT, TreatNaive, TreatmentIncluded, MSM)

write.table(Results, filename, append=TRUE, sep="|", row.names=FALSE, col.names=FALSE)

#Look at proportion treated based on E[Y=1|A=0]

for(t in seq(from=0.05, to=0.7, length=100)){

TreatmentDecision <- Performance3.Data %>%

mutate(SIT_Treated = ifelse(SIT>t, 1, 0),

TN_Treated = ifelse(TreatNaive>t, 1, 0),

TI_Treated = ifelse(TreatmentIncluded>t, 1, 0),

MSM_Treated = ifelse(MSM>t, 1, 0)) %>%

summarise(SIT = mean(SIT_Treated),

TN = mean(TN_Treated),

TI = mean(TI_Treated),

MSM = mean(MSM_Treated)) %>%

mutate(TreatmentThreshold = t,

Iteration = iter,

A0_X1_Value = gamma[g]) %>%

select(Iteration, A0_X1_Value, SIT, TN, TI, MSM, TreatmentThreshold)

write.table(TreatmentDecision, file = paste("TreatmentDecision_", filename, sep=""),

sep = "\t", append=TRUE, row.names = FALSE, col.names=FALSE)

}

info <- sprintf(paste("Simulation %d%% Completed"), round((iter/(iterations)*100)))

setWinProgressBar(pb, iter/((iterations))*100, label=info)

}

close(pb)

}

}

###-----------------------------------------------------------------------

## Data generating function

###-----------------------------------------------------------------------

Data.gen.fnc <- function(samplesize,

betaA0, betaA1, betaX0, betaX1,

phi, theta, gamma, tau,

Eventrate_A0,

Eventrate_A1,

Eventrate_Y,

SimulationSetting){

### Generate data

Data.gen <- data.frame("X0" = rnorm(samplesize, 0, 1))

alpha0 <- as.numeric(coef(glm(rbinom(samplesize, 1, Eventrate_A0)~offset(Data.gen$X0*phi),

family=binomial(link="logit")))) #control the proportion of treated at time 0

Data.gen <- Data.gen %>%

mutate(A0 = rbinom(samplesize, 1, exp(alpha0 + X0*phi)/(1+exp(alpha0 + X0*phi))),

X1 = rnorm(nrow(.),(X0 + (gamma*A0)), 1))

if(SimulationSetting=="RCT"){

if(theta<0 | theta>1){stop("For SimulationSetting=RCT, parameter theta must be between 0 and 1")}

Data.gen <- Data.gen %>%

mutate(A1 = rbinom(samplesize, 1, A0*theta))

}else{

alpha1 <- as.numeric(coef(glm(rbinom(samplesize, 1, Eventrate_A1)~offset(Data.gen$X1*phi + Data.gen$A0*theta +

Data.gen$X1*Data.gen$A0*tau),

family=binomial(link="logit")))) #control the proportion of treated at time 1

Data.gen <- Data.gen %>%

mutate(A1 = rbinom(samplesize, 1,

exp(alpha1 + X1*phi + A0*theta + X1*A0*tau)/(1+exp(alpha1 + X1*phi + A0*theta + X1*A0*tau))))

}

### Generate outcome data

alpha_Y <- as.numeric(coef(glm(rbinom(samplesize, 1, Eventrate_Y)~offset(Data.gen$X0*betaX0 + Data.gen$X1*betaX1 +

Data.gen$A0*betaA0 + Data.gen$A1*betaA1),

family=binomial(link="logit")))) #control the proportion of Y=1

Data.gen <- Data.gen %>%

mutate(Y = rbinom(samplesize, 1,

exp(alpha_Y + X0*betaX0 + X1*betaX1 + A0*betaA0 + A1*betaA1)/

(1+exp(alpha_Y + X0*betaX0 + X1*betaX1 + A0*betaA0 + A1*betaA1))))

return(list("Data.gen"=Data.gen,

"alpha_Y"=alpha_Y))

}

###-----------------------------------------------------------------------

## Data generating function - modified to fix all A0=A1=0 (i.e. no treatment)

###-----------------------------------------------------------------------

Modified.Data.gen.fnc <- function(samplesize,

betaA0, betaA1, betaX0, betaX1,

gamma,

alpha_y){

### Generate data

Data.gen <- data.frame("X0" = rnorm(samplesize, 0, 1)) %>%

mutate(A0 = rep(0, samplesize),

X1 = rnorm(samplesize,(X0 + (gamma*A0)), 1),

A1 = rep(0, samplesize),

Y = rbinom(samplesize, 1,

exp(alpha_y + X0*betaX0 + X1*betaX1 + A0*betaA0 + A1*betaA1)/

(1+exp(alpha_y + X0*betaX0 + X1*betaX1 + A0*betaA0 + A1*betaA1))))

return(Data.gen)

}

###-----------------------------------------------------------------------

## Marginal structure model function - returns standadised weights

###-----------------------------------------------------------------------

msm.fnc <- function(Dataset, Timepoints = 2){

numerator <- denominator <- matrix(NA, ncol=Timepoints, nrow=nrow(Dataset))

#Time zero

denominator.mod.T0 <- glm(A0 ~ X0, data=Dataset, family=binomial(link="logit"))

numerator.mod.T0 <- glm(A0 ~ X0, data=Dataset, family=binomial(link="logit"))

p_k <- predict(denominator.mod.T0, type="response")

p.star_k <- predict(numerator.mod.T0, type="response")

a_k <- c(Dataset$A0)

denominator[,1] <- (p_k^(a_k))*((1-p_k)^(1-a_k))

numerator[,1] <- (p.star_k^(a_k))*((1-p.star_k)^(1-a_k))

#Time one

denominator.mod.T1 <- glm(A1 ~ A0 + X0 + X1, data=Dataset, family=binomial(link="logit"))

numerator.mod.T1 <- glm(A1 ~ A0 + X0, data=Dataset, family=binomial(link="logit"))

p_k <- predict(denominator.mod.T1, type="response")

p.star_k <- predict(numerator.mod.T1, type="response")

a_k <- c(Dataset$A1)

denominator[,2] <- (p_k^(a_k))*((1-p_k)^(1-a_k))

numerator[,2] <- (p.star_k^(a_k))*((1-p.star_k)^(1-a_k))

#Calculate the weights

sw <- apply(numerator, 1, prod) / apply(denominator, 1, prod)

return(sw)

}

###-----------------------------------------------------------------------

## Manual weighted maximum likelihood

###-----------------------------------------------------------------------

# likelihood.fn <- function(W, y, LP, sw){

# #input: W = the vector of parameters

# # LP = the matrix of linear predictors from each simulated model

# # y = the vector of outcomes

# # sw = vector of standadized weights for each patient

#

# joint <- sum(-(sw*y*log(1+exp(-(LP%*%W))))-(sw*(1-y)*log(1+exp(LP%*%W)))) #log-Likelihood of logistic regression

#

# return(-(joint)) #optim minimises and so return minus log-likelihood in order to maximse

# }

# Weighted.Log.Reg <- function(LinPreds, Outcome, sw){

# #input: LinPreds = the matrix of linear predictors from each simulated model

# # Outcome = the vector of binary outcomes

# # sw = vector of standadized weights for each patient

#

# bl <- rep(-Inf, ncol(LinPreds)+1)

# bu <- rep(Inf, ncol(LinPreds)+1)

# start <- rep(0, ncol(LinPreds)+1)

#

# if(!is.matrix(LinPreds)){

# LinPreds <- data.matrix(LinPreds)

# }

#

# LinPreds <- cbind(rep(1, dim(LinPreds)[1]), LinPreds) #Add intercept into the design matrix

#

# MLE <- optim(start, likelihood.fn, y=Outcome, LP=LinPreds, sw=sw,

# method="L-BFGS-B", lower=bl, upper=bu, hessian=FALSE)

#

# return(MLE)

# }

###-----------------------------------------------------------------------

## Model performance function

###-----------------------------------------------------------------------

performance.fnc <- function(Outcomes, PredRisks, calcAUC=TRUE){

if(calcAUC){ #should the AUC be calculated?

Perform <- rep(NA, 4)

calinter <- glm(Outcomes~offset(log(PredRisks/(1-PredRisks))), family=binomial(link="logit"))

calslope <- glm(Outcomes~(log(PredRisks/(1-PredRisks))), family=binomial(link="logit"))

discrim <- roc(response=Outcomes, predictor=PredRisks, algorithm = 2, ci=FALSE, plot=FALSE)

BrierScore <- mean((PredRisks-Outcomes)^2)

Perform[1] <- coef(calinter)[1]

Perform[2] <- coef(calslope)[2]

Perform[3] <- as.numeric(discrim$auc)

Perform[4] <- BrierScore

names(Perform) <- c("CalInt", "CalSlope", "AUC", "BrierScore")

}else{

Perform <- rep(NA, 3)

calinter <- glm(Outcomes~offset(log(PredRisks/(1-PredRisks))), family=binomial(link="logit"))

calslope <- glm(Outcomes~(log(PredRisks/(1-PredRisks))), family=binomial(link="logit"))

BrierScore <- mean((PredRisks-Outcomes)^2)

Perform[1] <- coef(calinter)[1]

Perform[2] <- coef(calslope)[2]

Perform[3] <- BrierScore

names(Perform) <- c("CalInt", "CalSlope", "BrierScore")

}

return("Perform"=Perform)

}

###-----------------------------------------------------------------------

## Run the simulation

###-----------------------------------------------------------------------

#set working directory

setwd("~/Research/Treatment drop-in CPMs/Code")

gamma.vals <- c(-3, -2.5, -2, -1.5, -1, -0.5, 0)

sim.fnc(dev.samplesize = 10000, val.samplesize = 100000,

iterations = 1000, startingseed = 654673, filename = "RCT_NoDropOut",

betaA0 = log(0.5),

betaA1 = log(0.5),

betaX0 = log(1.5),

betaX1 = log(1.5),

phi = 0,

theta = 1,

gamma = gamma.vals,

tau = 0,

Eventrate_A0 = 0.5,

Eventrate_A1 = 0.5,

Eventrate_Y = 0.2,

calcAUC = TRUE,

SimulationSetting = "RCT")

sim.fnc(dev.samplesize = 10000, val.samplesize = 100000,

iterations = 1000, startingseed = 654673, filename = "RCT_10DropOut",

betaA0 = log(0.5),

betaA1 = log(0.5),

betaX0 = log(1.5),

betaX1 = log(1.5),

phi = 0,

theta = 0.9,

gamma = gamma.vals,

tau = 0,

Eventrate_A0 = 0.5,

Eventrate_A1 = 0.5,

Eventrate_Y = 0.2,

calcAUC = TRUE,

SimulationSetting = "RCT")

sim.fnc(dev.samplesize = 10000, val.samplesize = 100000,

iterations = 1000, startingseed = 654673, filename = "Observational_50Treated",

betaA0 = log(0.5),

betaA1 = log(0.5),

betaX0 = log(1.5),

betaX1 = log(1.5),

phi = log(2),

theta = log(2),

gamma = gamma.vals,

tau = 0,

Eventrate_A0 = 0.5,

Eventrate_A1 = 0.5,

Eventrate_Y = 0.2,

calcAUC = TRUE,

SimulationSetting = "Observational")

sim.fnc(dev.samplesize = 10000, val.samplesize = 100000,

iterations = 1000, startingseed = 654673, filename = "Observational_20Treated",

betaA0 = log(0.5),

betaA1 = log(0.5),

betaX0 = log(1.5),

betaX1 = log(1.5),

phi = log(2),

theta = log(2),

gamma = gamma.vals,

tau = 0,

Eventrate_A0 = 0.2,

Eventrate_A1 = 0.2,

Eventrate_Y = 0.2,

calcAUC = FALSE,

SimulationSetting = "Observational")

###-----------------------------------------------------------------------

## Explore the results

###-----------------------------------------------------------------------

library(plyr)

library(dplyr)

library(reshape2)

library(ggplot2)

library(cowplot)

RCT_NoDropOut_simulationresults <- read.table("RCT_NoDropOut.txt", header=TRUE, sep = "|") %>%

melt(id.vars=c("Iteration", "A0_X1_Value", "Estimand"), variable.name ="Model") %>%

mutate(SimulationScenario = "RCT: no drop out")

RCT_10DropOut_simulationresults <- read.table("RCT_10DropOut.txt", header=TRUE, sep = "|") %>%

melt(id.vars=c("Iteration", "A0_X1_Value", "Estimand"), variable.name ="Model") %>%

mutate(SimulationScenario = "RCT: 10% drop-out")

Observational_50Treated_simulationresults <- read.table("Observational_50Treated.txt", header=TRUE, sep = "|") %>%

melt(id.vars=c("Iteration", "A0_X1_Value", "Estimand"), variable.name ="Model") %>%

mutate(SimulationScenario = "Observational: 50% treated")

Observational_20Treated_simulationresults <- read.table("Observational_20Treated.txt", header=TRUE, sep = "|") %>%

melt(id.vars=c("Iteration", "A0_X1_Value", "Estimand"), variable.name ="Model") %>%

mutate(SimulationScenario = "Observational: 20% treated")

SimulationResults <- RCT_NoDropOut_simulationresults %>%

bind_rows(RCT_10DropOut_simulationresults) %>%

bind_rows(Observational_50Treated_simulationresults) %>%

bind_rows(Observational_20Treated_simulationresults)

SimulationResults$SimulationScenario <- factor(SimulationResults$SimulationScenario,

levels = c("RCT: no drop out", "RCT: 10% drop-out",

"Observational: 50% treated", "Observational: 20% treated"))

Plot.data <- SimulationResults %>%

group_by(A0_X1_Value, Model, Estimand, SimulationScenario) %>%

summarise(est = mean(value),

SE = sd(value)) %>%

mutate(CI_lower = est-(1.96*(SE)),

CI_Upper = est+(1.96*(SE)),

Type = factor(ifelse(grepl("Performance.E1", Estimand), "Performance-setting: MT",

ifelse(grepl("Performance.E2", Estimand), "Performance-setting: NBT",

"Performance-setting: NTT")))) %>%

ungroup() %>%

select(-SE) %>%

mutate(Model = revalue(Model, c(SIT="Ignore Treatment",

TreatNaive = "Treatment-Naive",

TreatmentIncluded = "Model Treatment",

MSM = "MSM")))

##Calibration Intercept

CalInt <- ggplot(Plot.data %>% filter(Estimand=="Performance.E1_CalInt" |

Estimand=="Performance.E2_CalInt" |

Estimand=="Performance.E3_CalInt") %>%

filter(SimulationScenario!="RCT: no drop out"),

aes(x=A0_X1_Value, y=est, group=Model, colour=Model)) +

geom_line() +

geom_ribbon(aes(ymin=CI_lower, ymax=CI_Upper, fill=Model), alpha = 0.3, colour=NA)+

facet_grid(SimulationScenario~Type, scales="fixed") +

ylab("Calibration Intercept") + xlab(expression(gamma)) +

theme_bw(base_size = 12) +

theme(legend.text=element_text(size=12), legend.position="top", legend.title=element_blank()) +

scale_colour_manual(values=c("#E69F00", "#D55E00", "#009E73", "#0072B2")) +

scale_fill_manual(values=c("#E69F00", "#D55E00", "#009E73", "#0072B2"))

##Calibration Slope

CalSlope <- ggplot(Plot.data %>% filter(Estimand=="Performance.E1_CalSlope" |

Estimand=="Performance.E2_CalSlope" |

Estimand=="Performance.E3_CalSlope") %>%

filter(SimulationScenario!="RCT: no drop out"),

aes(x=A0_X1_Value, y=est, group=Model, colour=Model)) +

geom_line() +

geom_ribbon(aes(ymin=CI_lower, ymax=CI_Upper, fill=Model), alpha = 0.3,colour=NA)+

facet_grid(SimulationScenario~Type, scales="fixed") +

ylab("Calibration Slope") + xlab(expression(gamma)) +

theme_bw(base_size = 12) +

theme(legend.text=element_text(size=12), legend.position="top", legend.title=element_blank()) +

#theme(legend.position="none") +

scale_colour_manual(values=c("#E69F00", "#D55E00", "#009E73", "#0072B2")) +

scale_fill_manual(values=c("#E69F00", "#D55E00", "#009E73", "#0072B2"))

plot_grid(CalInt, CalSlope, align = "v", nrow=2, ncol=1, rel_heights=c(1,0.85))

##AUC

AUC <- ggplot(Plot.data %>% filter(Estimand=="Performance.E1_AUC" |

Estimand=="Performance.E2_AUC" |

Estimand=="Performance.E3_AUC") %>%

filter(SimulationScenario=="RCT: 10% drop-out"),

aes(x=A0_X1_Value, y=est, group=Model, colour=Model)) +

geom_line() +

geom_ribbon(aes(ymin=CI_lower, ymax=CI_Upper, fill=Model), alpha = 0.3, colour=NA)+

facet_wrap(~Type, ncol=3, scales="fixed") +

ylab("AUC") + xlab(expression(gamma)) +

theme_bw(base_size = 12) +

theme(legend.text=element_text(size=12), legend.position="top") +

scale_colour_manual(values=c("#E69F00", "#D55E00", "#009E73", "#0072B2")) +

scale_fill_manual(values=c("#E69F00", "#D55E00", "#009E73", "#0072B2"))

##Brier Score

BS <- ggplot(Plot.data %>% filter(Estimand=="Performance.E1_BrierScore" |

Estimand=="Performance.E2_BrierScore" |

Estimand=="Performance.E3_BrierScore") %>%

filter(SimulationScenario=="RCT: 10% drop-out"),

aes(x=A0_X1_Value, y=est, group=Model, colour=Model)) +

geom_line() +

geom_ribbon(aes(ymin=CI_lower, ymax=CI_Upper, fill=Model), alpha = 0.3, colour=NA)+

facet_wrap(~Type, ncol=3, scales="fixed") +

ylab("Brier Score") + xlab(expression(gamma)) +

theme_bw(base_size = 12) +

#theme(legend.text=element_text(size=12), legend.position="top", legend.title=element_blank()) +

theme(legend.position="none") +

scale_colour_manual(values=c("#E69F00", "#D55E00", "#009E73", "#0072B2")) +

scale_fill_manual(values=c("#E69F00", "#D55E00", "#009E73", "#0072B2"))

plot_grid(AUC, BS, align = "v", nrow=2, ncol=1, rel_heights=c(1,0.85))

###-----------------------------------------------------------------------

## Explore the Treatment decision results

###-----------------------------------------------------------------------

library(plyr)

library(dplyr)

library(reshape2)

library(ggplot2)

library(cowplot)

TreatmentDecision <- read.table("TreatmentDecision_Observational_50Treated.txt", header=TRUE, sep = "\t") %>%

group_by(A0_X1_Value, TreatmentThreshold) %>%

summarise(SIT = mean(SIT),

TN = mean(TN),

TI = mean(TI),

MSM = mean(MSM)) %>%

melt(id.vars=c("A0_X1_Value", "TreatmentThreshold")) %>%

ungroup() %>%

mutate(variable = revalue(variable, c(SIT="Ignore Treatment",

TN = "Treatment-Naive",

TI = "Model Treatment",

MSM = "MSM")),

A0_X1_Value = factor(A0_X1_Value,

levels=unique(A0_X1_Value),

labels=c(paste("gamma", "==", unique(A0_X1_Value)))))

ggplot(TreatmentDecision %>% filter(A0_X1_Value=="gamma == -3" |

A0_X1_Value=="gamma == -2" |

A0_X1_Value=="gamma == -1" |

A0_X1_Value=="gamma == 0"),

aes(x=TreatmentThreshold, y=value, group=variable, colour=variable)) +

geom_line() +

facet_wrap(~A0_X1_Value, ncol=2, scales="fixed", labeller=label_parsed) +

ylab("Proportion Treated") + xlab("Treatment Threshold") +

theme_bw(base_size = 12) +

theme(legend.text=element_text(size=12), legend.position="top", legend.title=element_blank()) +

scale_colour_manual(values=c("#E69F00", "#D55E00", "#009E73", "#0072B2")) +

scale_fill_manual(values=c("#E69F00", "#D55E00", "#009E73", "#0072B2"))

ggsave("TreatmentDecision_Observational_50Treated.tiff", height = 6, width = 6, units = "in", dpi=300)
